# A Cautionary Report for Pathogen Identification Using Shotgun Metagenomics; A Comparison to Aerobic Culture and Polymerase Chain Reaction for *Salmonella enterica* Identification

**DOI:** 10.3389/fmicb.2019.02499

**Published:** 2019-11-01

**Authors:** Enrique Doster, Pablo Rovira, Noelle R. Noyes, Brandy A. Burgess, Xiang Yang, Margaret D. Weinroth, Lyndsey Linke, Roberta Magnuson, Christina Boucher, Keith E. Belk, Paul S. Morley

**Affiliations:** ^1^Department of Microbiology, Immunology and Pathology, Colorado State University, Fort Collins, CO, United States; ^2^Instituto Nacional de Investigacion Agropecuaria, Treinta y Tres, Uruguay; ^3^Department of Veterinary Population Medicine, University of Minnesota, St. Paul, MN, United States; ^4^Department of Population Health, University of Georgia, Athens, GA, United States; ^5^Department of Animal Science, University of California, Davis, Davis, CA, United States; ^6^Department of Animal Sciences, Colorado State University, Fort Collins, CO, United States; ^7^Department of Clinical Sciences, Colorado State University, Fort Collins, CO, United States; ^8^Department of Computer and Information Science and Engineering, University of Florida, Gainesville, FL, United States; ^9^Veterinary Education, Research, and Outreach Center, West Texas A&M University, Canyon, TX, United States

**Keywords:** shotgun metagenomics, *Salmonella enterica*, culture, PCR, pathogen identification

## Abstract

This study was conducted to compare aerobic culture, polymerase chain reaction (PCR), lateral flow immunoassay (LFI), and shotgun metagenomics for identification of *Salmonella enterica* in feces collected from feedlot cattle. Samples were analyzed in parallel using all four tests. Results from aerobic culture and PCR were 100% concordant and indicated low *S. enterica* prevalence (3/60 samples positive). Although low *S. enterica* prevalence restricted formal statistical comparisons, LFI and deep metagenomic sequencing results were discordant with these results. Specifically, metagenomic analysis using *k*-mer-based classification against the RefSeq database indicated that 11/60 of samples contained sequence reads that matched to the *S. enterica* genome and uniquely identified this species of bacteria within the sample. However, further examination revealed that plasmid sequences were often included with bacterial genomic sequence data submitted to NCBI, which can lead to incorrect taxonomic classification. To circumvent this classification problem, we separated all plasmid sequences included in bacterial RefSeq genomes and reassigned them to a unique taxon so that they would not be uniquely associated with specific bacterial species such as *S. enterica*. Using this revised database and taxonomic structure, we found that only 6/60 samples contained sequences specific for *S. enterica*, suggesting increased relative specificity. Reads identified as *S. enterica* in these six samples were further evaluated using BLAST and NCBI’s nr/nt database, which identified that only 2/60 samples contained reads exclusive to *S. enterica* chromosomal genomes. These two samples were culture- and PCR-negative, suggesting that even deep metagenomic sequencing suffers from lower sensitivity and specificity in comparison to more traditional pathogen detection methods. Additionally, no sample reads were taxonomically classified as *S. enterica* with two other metagenomic tools, Metagenomic Intra-species Diversity Analysis System (MIDAS) and Metagenomic Phylogenetic Analysis 2 (MetaPhlAn2). This study re-affirmed that the traditional techniques of aerobic culture and PCR provide similar results for *S. enterica* identification in cattle feces. On the other hand, metagenomic results are highly influenced by the classification method and reference database employed. These results highlight the nuances of computational detection of species-level sequences within short-read metagenomic sequence data, and emphasize the need for cautious interpretation of such results.

## Introduction

The study and detection of microbial organisms has long been reliant on cultivation and characterization of certain species, but advancements in sequencing technologies have revealed an underlying microbial diversity largely ignored by culture-based techniques (Staley and Konopka, 1985; Hugenholtz, 2002; Stewart et al., 2018). High-throughput sequencing techniques now enable a culture-independent metagenomic approach that provides access to DNA from all bacteria (microbiome) within a given sample. This rapidly developing technology provides great potential for investigating the complexity of bacterial communities (Turnbaugh et al., 2007; Forsberg et al., 2012; Noyes et al., 2016). However, there are limited numbers of investigations evaluating the relationship between metagenomic results and traditional diagnostic methods. Metagenomic approaches have been used to find novel pathogens when traditional methods were not fruitful (Gire et al., 2014; Zhou et al., 2016), but the relative increased sensitivity in metagenomic approaches raises important questions about their use in identification of foodborne pathogens in fecal samples. One such example is *Salmonella enterica*, an important zoonotic pathogen that causes over 93 million cases of gastroenteritis in humans globally every year (Majowicz et al., 2010) and has been implicated in outbreaks associated with beef products (Laufer et al., 2015). Accurate identification and characterization of *S. enterica* is critical for improving food safety and preventing foodborne disease outbreaks. Therefore, utilizing samples obtained from another study of feedlot cattle (Doster et al., 2018), we compared a metagenomic approach for *S. enterica* identification to the traditional techniques of aerobic culture, polymerase chain reaction (PCR), and lateral flow immunoassays (LFI).

## Materials and Methods

### Sample Processing

This study compared aerobic culture, PCR, LFIs, and shotgun metagenomic sequencing for *S. enterica* identification in fecal samples collected from feedlot cattle. These samples are from a published investigation on the effect of metaphylactic treatment with tulathromycin (one of the most commonly used antimicrobial drugs in American beef feedlots) on the resistome and microbiome of feedlot cattle (Doster et al., 2018). As previously described, two groups of cattle originating from a single facility were enrolled into the study before their arrival at a commercial cattle feedlot in Texas and individually sampled during arrival processing at the feedlot and 11 days later. A total of 346 samples collected during arrival processing were analyzed with aerobic culture for *S. enterica* identification. Three samples of those collected at arrival were culture-positive for *S. enterica* (0.87% prevalence, 3/346). Budgetary limitations for shotgun metagenomic sequencing and PCR testing did not allow evaluation of all cattle sampled at arrival, therefore a subset of 30 cattle were sampled 11 days later and their corresponding arrival samples were included in this study (*N* = 60).

All fecal samples underwent nutrient enrichment and aerobic culture with tetrathionate broth (9:1 broth volume:fecal mass; Difco Laboratories, Sparks, MD, United States), Rappaport-Vassiliadis R10 broth (Difco Laboratories), and streaked for isolation on xylose-lysine-tergitol (XLT-4) agar plates (BD Diagnostic Systems, Sparks, MD, United States). Single colonies were arbitrarily chosen for isolation on tryptic soy agar with 5% sheep’s blood (TSA) (BBL, Sparks, MD, United States) from each sample containing colonies with characteristics indicative of *S. enterica* (smooth, round, black colonies). Individual colonies were then classified to five different common serogroups (B, C1, C2, D, E, and K) with polyvalent O antiserum (Difco Laboratories) for Salmonella serogroup confirmation. Additionally, for each sample during aerobic culture, LFIs strips (Reveal 2.0, NEOGEN, Lansing, MI, United States) were used to test the tetrathionate broth for *S. enterica* identification. These strips have been tested with horse fecal samples and show promising sensitivity and specificity for rapid identification of *S. enterica* (Burgess et al., 2015, 2014), but have not been tested extensively in cattle feces. The 60 samples selected for metagenomic sequencing were also tested by qPCR for *S. enterica* detection (Applied Biosystems, Foster City, CA, United States) using a 2 ml aliquot of enriched culture media (TET).

Fecal samples selected for metagenomic sequencing were processed for DNA extraction and metagenomic sequencing. After removal of excess plant debris and reduction of inhibitors, total DNA was extracted from each sample using the PowerMax Soil DNA Isolation Kit (MO BIO Laboratories) following the manufacturer’s protocol. DNA concentration and quality were evaluated using a NanoDrop^TM^ spectrophotometer (Thermo Fisher Scientific, Inc.). Samples with 260 nm:280 nm ratios >1.3 and DNA concentrations >20 ng/μl were sent for sequencing; samples that did not meet these thresholds were concentrated by ethanol precipitation before sequencing. 100 μl aliquots of purified DNA from all 60 samples were delivered to the Genomics and Microarray Core at University of Colorado Denver for library preparation and sequencing (Aurora, CO). Genomic libraries were prepared using the TruSeq DNA Library Preparation Kit (Illumina, Inc.). Next-generation sequencing was completed on the HiSeq 2000 (Illumina, Inc.) with five samples per lane, V4 chemistry, and paired-end reads of 125 bp in length.

### Trimming and Filtering of Metagenomic Sequence Data

De-multiplexed sequence reads from libraries sequenced on the HiSeq 2000 were processed using the AMRPlusPlus bioinformatic pipeline (Lakin et al., 2017). Briefly, the Trimmomatic software (Bolger et al., 2014) was used to remove low quality sequences, and the “ILLUMINACLIP” command was employed to remove Illumina TruSeq adapters added during library preparation. Sample reads were filtered to remove sequences mapping to the reference *Bos taurus* genome (Merchant et al., 2014). Sequencing results resulting from the number of raw, trimmed, and filtered reads and the average Phred score for each sample were compared using the generalized linear models with the “glm” function and the R platform (R Development Core Team, 2008) to assess systematic sequencing bias across sequencing batches. Similarly, differences in sequencing results between sample groups were tested with the Wilcoxon signed–rank test when comparing paired values from the same animal (arrival to day 11) and the Wilcoxon rank–sum test was employed when comparing animals at either time point.

### Microbiome – Classification of Bacterial Sequences and Identification of *Salmonella enteric*a

Kraken 2 (Wood et al., 2019) was used to assign taxonomic labels to shotgun metagenomic DNA sequences using the National Center for Biotechnology Information’s (NCBI) (NCBI Resource Coordinators, 2016) Reference Sequence Database (RefSeq) (O’Leary et al., 2016). RefSeq represents the most comprehensive, integrated, well-annotated set of genomes that includes viruses, archaea, and bacteria. Kraken 2 takes the metagenomic sample reads that are typically 125 nucleotides each, partition the reads into 31 nucleotide long pieces, and searches for exact matches to the RefSeq reference database. Every match is scored with Kraken 2’s lowest-common-ancestor algorithm and the read is classified to the taxonomic level with the most points. The number of samples with reads classified as *S. enterica* were identified and sample prevalence results were compared in contingency tables for shotgun metagenomics, aerobic culture and PCR. Reads classified as *S. enterica* were re-classified using the complete NCBI’s nr/nt database using BLAST. Results suggested that plasmid sequences were being misclassified as *S. enterica* so to increase classification accuracy, we created a Kraken 2 “modified database” consisting of curated bacteria, archaea, and viral genomes from RefSeq for a total of 19,919 genomes. To increase the specificity of taxonomic read classification and account for the horizontal transfer of plasmids in microbial communities, the term “plasmid” was searched for using the command-line tool, “sed”, and each header was modified to be associated with the taxa ID for “unidentified plasmid”, NCBI:txid 45202^1^. Plasmids were identified in 11.5% (2,291/19,919) of the genomes within RefSeq. Kraken 2’s highest confidence value of “1” was selected to increase the alignment score threshold required for species-level classification and increase the accuracy of classification at higher taxonomic levels. To further improve specificity, reads classified as *S. enterica* were re-classified with BLAST and NCBI’s nr/nt database to confirm that sequences are truly unique to *S. enterica* when considering all available sequences on NCBI^2^. Additionally, sample reads were taxonomically classified with two other tools using standard settings: Metagenomic Intra-species Diversity Analysis System (MIDAS) and Metagenomic Phylogenetic Analysis 2 (MetaPhlAn2). Results from both tools were interrogated to identify the number of samples with reads mapping to *S. enterica*.

## Results

### Sample Collection, Culture, Lateral Flow Immunoassay, and PCR Results

Aerobic culture and LFIs were used to test for the presence *of S. enterica* on 376 fecal samples collected from study cattle. Aerobic culture identified three positive samples in the sample set included in this study, and agglutination tests revealed that two isolates recovered from arrival processing samples were serogroup C1 (including from the animal unsuccessfully sampled on day 11) and another serogroup K isolate, while the isolate recovered on day 11 was serogroup C1. These same three samples were positive when tested with PCR, suggesting a 5% (3/60) overall prevalence for *S. enterica* shedding during the study period (Table 1). There were 63 of 376 fecal samples that were positive using the commercial LFI assay, none of which were culture- or PCR-positive, indicating that this test is inappropriate for classification of *S. enterica* fecal shedding status in feedlot cattle.

**TABLE 1:** Aerobic culture, PCR, and lateral flow immunoassay (LFI) results for *Salmonella enterica* identification in 60 fecal samples collected from feedlot cattle.

	**Culture and PCR**		
**Lateral Flow**	**Positive**	**Negative**	**Total**
Positive	0	13	13
Negative	3	44	47
Total	3	57	60

### Sequencing Results

Shotgun metagenomic sequencing generated 5.89 billion reads (2.95 billion paired reads) across 60 samples with an average of 98.20 million reads per sample [range 26.98 – 160.71 M (Supplementary Table S1)]. The average Phred quality score of raw reads across all samples was 35.2 (range 34.54 – 35.82). Because of the high average Phred scores across samples, only 3.82% of reads were removed for low quality (minimum per sample = 2.21%, maximum = 6.36%). Of the remaining reads, 0.03% (1.8 M reads) were identified as bovine DNA and removed from subsequent analysis; two samples were nearly 20% bovine DNA and the other 58 samples ranged from 0.03 to 4.57%. Overall, there was no evidence of systematic bias in the sequencing results among samples.

### Identification of *Salmonella enterica* Using Shotgun Metagenomics

**FIGURE 1:**
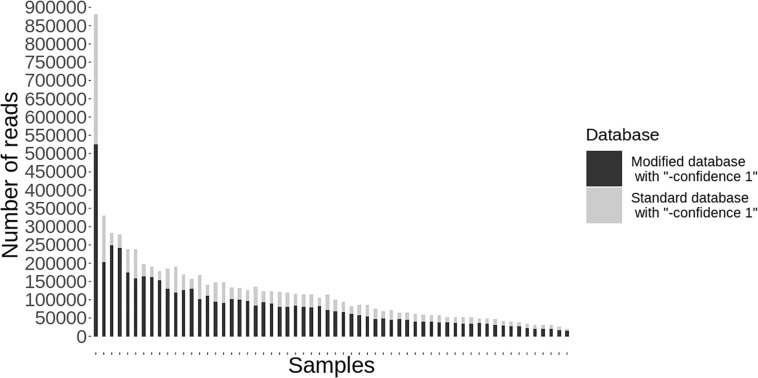
Individual samples (*n* = 60) on the *x*-axis with the total number of reads classified taxonomically using kraken 2 on the *y*-axis. Reads classified using the standard database are shown in gray and the decreased number of reads classified using the modified database are shown in black.

Following quality-based read trimming and removal of host genetic contamination, the Kraken 2 software (Wood et al., 2019) was used to classify shotgun metagenomic reads taxonomically with NCBI’s RefSeq database. The Kraken 2 flag, “–confidence” was used with the highest value of “1” to increase the score required to meet the threshold for species level classification. On average, 99.8% of the reads in each sample were unclassified (minimum 97.89%, maximum 99.91%). In all, more than 7.3 million reads were taxonomically classified with an average of 122,900 reads per sample. Using Kraken 2 to analyze these data, *S. enterica* was identified in 18.3% (11/60) of samples, compared to 5% prevalence using culture or PCR (Supplementary Table S1). However, through further examination of the RefSeq database structure, we noted that plasmid sequences, which can be actively transferred between bacteria, are commonly included within the reference genome files for each species. Kraken 2 then incorrectly classifies these plasmids as being conserved (species-specific) to the organism that carried the plasmid when it was sequenced and submitted to NCBI. Therefore, we modified the creation of the Kraken 2 database by separating the plasmid sequences included with RefSeq genomes and re-assigning them to a single taxon for all plasmid and synthetic vector sequences. Following re-classification of reads with the modified database, only 10% (6/60) of samples were *S. enterica-*positive, suggesting increased relative specificity compared to the standard database (Table 2). The number of misclassified reads as plasmids, on average, made up 16.6% of each sample’s total reads classified using Kraken 2 (Figure 1). As a point of comparison, we further assessed how modifying the plasmid sequences affected the reported prevalence for *Campylobacter jejuni*, *Enterococcus faecalis*, and *Escherichia coli* in the metagenomic data. In general, use of the modified database decreased the number of samples with reads mapping to species of interest compared to the standard database, but the magnitude of this difference varied by species (Table 3). If the “–confidence 1” flag was not used (i.e., if default Kraken 2 settings were used), all four species were positively identified in all 60 samples (Supplementary Table S2).

**TABLE 2:** Aerobic culture and PCR (100% agreement) compared to shotgun metagenomic analysis with Kraken 2 for *Salmonella enterica* identification in 60 samples.

	**Culture and PCR**		
**Kraken 2 standard db**	**Positive**	**Negative**	**Total**
Positive	1	10	11
Negative	2	47	49
Total	3	57	60
**Kraken 2 modified db**			
Positive	1	5	6
Negative	2	52	54
Total	3	57	60
**Kraken 2 modified db and BLAST confirmation**			
Positive	0	2	2
Negative	3	55	58
Total	3	57	60

*Results are compared between using the standard Kraken 2 database, a modified Kraken 2 database which considers plasmid sequences as unique taxa, and the modified database with additional confirmation using BLAST and NCBI’s nr/nt database. Kraken 2 was run with the “–confidence” flag at the highest value, “1”.*

**TABLE 3:** Number of samples with at least 1 read mapping to *Salmonella enterica*, *Campylobacter jejuni, Enterococcus faecalis*, and *Escherichia coli* according the Kraken 2 database and “-confidence 1” flag used.

**Database**	***Salmonella enterica***	***Campylobacter jejuni***	***Enterococcus faecalis***	***Escherichia coli***
Standard	60	60	60	60
Standard with confidence “1”	11	59	40	59
Modified	60	60	60	60
Modified with confidence “1”	6	29	40	58

To further investigate the specificity of species level identification with the modified database, all sequences classified as *S. enterica* were isolated, and classification was confirmed using BLAST (Madden, 2013) version 2.8.1 and NCBI’s largest database of genetic sequences, nucleotide collection (nr/nt) (Supplementary Table S3). Out of the six samples that were positive for *S. enterica* based on Kraken 2, only two contained sequences that were confirmed to be unique to *S. enterica.* Notably, these results were not concordant with the positive culture and PCR results (Table 2). The remaining reads were classified to multiple different species with 99% sequence identity, but there was no evidence of misclassification above the family level, i.e., Enterobacteriaceae. The MIDAS and MetaPhlAn2 metagenomic classification tools did not identify any samples as containing *S. enterica*.

## Discussion

This study demonstrates that metagenomic sequencing results are not comparable to those from culture or PCR for species-level identification of *S. enterica* in fecal samples collected from feedlot cattle. While metagenomics greatly expands our ability to probe microbial ecology, it is important to understand the current limitations of this approach for applied uses such as pathogen detection. This is especially true given the need for species- or even strain-level classification to make relevant inferences regarding the vast majority of pathogens. Metagenomics will inevitably fit a complementary role in pathogen detection and surveillance as sequencing costs decrease, reference databases improve, and bioinformatic analyses are streamlined (Miller et al., 2013). However, results from this study illustrate that metagenomic approaches are reliant on validated use of bioinformatic methods, availability of extensive databases, and presence of uniquely identifying genetic sequences within the taxonomic tree; or alternatively use of long-read sequencing technology for metagenomic samples. Furthermore, we demonstrate that even deep shotgun sequencing of metagenomic samples does not provide sensitivity comparable to that of PCR and culture for a specific pathogen, a finding that has been consistently reported for low-abundance features within microbial communities (Noyes et al., 2017; Weinroth et al., 2018; Zaheer et al., 2018).

Aerobic culture and PCR are the most commonly used techniques for *S. enterica* identification in clinical and research settings, and these tests provided 100% concordant results in this study (Table 1). Using these concordant results, *S. enterica* prevalence in this sample set would have been reported at 5% (3/60 samples). By comparison, standard metagenomic analysis would have suggested the presence of *S. enterica* DNA in 100% (60/60) of samples, which could be misconstrued to signify an overabundance of foodborne pathogens in the beef production system. Only by progressively increasing the stringency required for species-level classification did we witness a decrease in the percentage of *S. enterica* positive samples; with the most strict parameters, 18.3% (11/60) of samples were classified as *S. enterica*-positive. Importantly, these 11 samples and these results were 18.1% discordant with PCR/culture. Next, we identified that plasmid sequences were also creating false-positive *S. enterica* identification. By removing these plasmid sequences during database creation, specificity was improved with only 10% (6/60) positive samples and 10% discordant results with PCR/culture. Finally, we performed additional bioinformatic analysis using a more comprehensive reference database (i.e., NCBI nr/nt) to confirm that only two of these six samples contained sequences truly unique to *S. enterica*. This final analysis produced *S. enterica* prevalence estimates closer to those obtained from aerobic culture and PCR (i.e., ∼5%), however, the results were 100% discordant with those tests. Thus, metagenomic analysis not only identified *S. enterica* DNA in samples that were *S. enterica* negative via culture and PCR, but also failed to identify *S. enterica* DNA in the three samples that were positive via culture and PCR. These results suggest that even deep metagenomic sequencing (i.e., ∼100 M paired-end reads per sample) is not only less specific than PCR and culture, but also less sensitive – a finding that was further buttressed by failure to identify *S. enterica* using both MetaPhlAn2 and MIDAS metagenomic pipelines.

While most bioinformatic pipelines enable user-defined parameter setting, numerous studies have shown that optimal parameters are often specific to a given sample type or even set of samples (Siegwald et al., 2017; Vollmers et al., 2017; Gardner et al., 2019). Without a known “truth status” for each sample, it is typically impossible to determine which combination of settings yield the most accurate results. Indeed, in this study we demonstrated that rational parameter setting was only possible through comparison with paired culture and PCR results from the same samples; without prior knowledge regarding *S. enterica* sample status, we would not have known whether bioinformatic parameters were too strict or too loose. Indeed, the lack of culture- or PCR-generated results for *C. jejuni*, *E. faecium*, and *E. coli* precludes any in-depth discussion about whether the metagenomic results obtained for these species were accurate.

A major impediment to achieving high specificity from short-read metagenomic data is the need to rely on incomplete reference databases, i.e., databases that do not encompass all known genomic sequences. Current metagenomic identification methods such as Kraken 2 rely on the presence of stretches of genomic DNA that uniquely discriminate between different bacterial taxa. However, this “uniqueness” is inherently defined by the set of DNA being included in the reference database. If the database is expanded, our previous notion of “uniqueness” may be disabused; in other words, the sequence that we thought was unique to *S. enterica* may actually be shared between multiple species, and thus can no longer be utilized as a discriminatory marker for *S. enterica*. This is the phenomenon we leveraged in order to demonstrate that the sequences being identified by Kraken 2 as *S. enterica* were actually shared with other bacterial species.

Achieving accurate and biologically relevant results from metagenomic analysis poses a challenge and opportunity to the scientific community, particularly when these results rely on sensitive and specific detection of species-level microbes. In contrast to ecological studies of microbial communities (in which shifts at the phylum level can be meaningful), pathogen detection typically necessitates accurate identification at the species (or even strain) level. This application pushes the limits of short-read metagenomic data and current bioinformatic tools (including reference databases), and therefore scientists must intensely scrutinize pathogen detection results obtained from metagenomic data. This scrutiny includes fulsome discussion of the full range of possible reasons for why the results may or may not be valid. This point is especially salient as the research, medical and regulatory communities continue to discuss application of shotgun metagenomics for purposes of disease diagnosis, outbreak investigation, and pathogen detection across a variety of environments. The possibilities of metagenomic data must not be allowed to overshadow the methodical yet critically important requirements of the scientific approach. As this work demonstrates, our ability to merge highly innovative methods with practical applications will depend on successful cooperation between scientists studying bacteria with traditional methods, those experimenting with a metagenomics approach, and scientists developing bioinformatic tools.

## Data Availability Statement

The metagenomic datasets generated for this study can be found in the National Center for Biotechnology Information’s collection of biological data, BioProject Accession PRJNA309291 (https://www.ncbi.nlm.nih.gov/bioproject/?term=309291).

## Ethics Statement

This study was reviewed and approved by the Colorado State University’s Research Integrity and Compliance Review Office (#2015-002).

## Author Contributions

KB and PM designed this study, obtained funding, secured partnerships with industry partners where the study was conducted, and provided oversight for all other aspects of the study. ED and PR administered experimental treatment and collected the samples. ED, PR, BB, XY, MW, LL, and RM performed the sample processing and laboratory testing. PR, NN, and CB oversaw bioinformatic analysis and interpretation of results. ED performed the bioinformatic analysis and drafting of the manuscript. All authors read, edited, and approved the final manuscript.

## Conflict of Interest

The authors declare that the research was conducted in the absence of any commercial or financial relationships that could be construed as a potential conflict of interest.

## Footnotes

1 https://github.com/colostatemeg/meglab-kraken-custom-db

2 https://github.com/EnriqueDoster/MEG-kraken-species-ID

## Supplementary Material

TABLE S1 Sample metadata, culture, and PCR results.

TABLE S2 Metagenomic count of reads classified as *Salmonella enterica*, *Campylobacter jejuni, Enterococcus faecalis*, and *Escherichia coli*.

TABLE S3 Metagenomic count of reads classified as *Salmonella enterica* and additional blast confirmation.
